# Microstructure Evolution, Hot Deformation Behavior and Processing Maps of an FeCrAl Alloy

**DOI:** 10.3390/ma17081847

**Published:** 2024-04-17

**Authors:** Xiang-Qian Fang, Jin-Bin Wang, Si-You Liu, Jun-Zhe Wen, Hong-Yu Song, Hai-Tao Liu

**Affiliations:** 1 State Key Laboratory of Rolling and Automation, School of Materials Science and Engineering, Northeastern University, Shenyang 110819, China; fangxiangqianneu@163.com (X.-Q.F.); wjb0226@sina.com (J.-B.W.); 2 School of Materials Science and Engineering, Northeastern University, Shenyang 110819, China; 20223899@stu.neu.edu.cn (S.-Y.L.); 20223911@stu.neu.edu.cn (J.-Z.W.)

**Keywords:** FeCrAl alloy, flow stress, constitutive equation, microstructure, hot processing map

## Abstract

The deteriorated plasticity arising from the insoluble precipitates may lead to cracks during the rolling of FeCrAl alloys. The microstructure evolution and hot deformation behavior of an FeCrAl alloy were investigated in the temperature range of 750–1200 °C and strain rate range of 0.01–10 s^−1^. The flow stress of the FeCrAl alloy decreased with an increasing deformation temperature and decreased strain rate during hot working. The thermal deformation activation energy was determined to be 329.49 kJ/mol based on the compression test. Then, the optimal hot working range was given based on the established hot processing maps. The hot processing map revealed four small instability zones. The optimal working range for the material was identified as follows: at a true strain of 0.69, the deformation temperature should be 1050–1200 °C, and the strain rate should be 0.01–0.4 s^−1^. The observation of key samples of thermally simulated compression showed that discontinuous dynamic recrystallization started to occur with the temperate above 1000 °C, leading to bended grain boundaries. When the temperature was increased to 1150 °C, the dynamic recrystallization resulted in a microstructure composed of fine and equiaxed grains.

## 1. Introduction

Fuel cladding prevents radioactive fission products from escaping the fuel matrix into the reactor coolant and contaminating it, which is one of the key aspects of the safety of the nuclear power plant. Thus, it needs a corrosion-resistant material with a low absorption cross section for thermal neutrons, and the Zirconium alloy is normally used as fuel cladding. However, the rapid reaction between this alloy and the water vapor may cause leakage and explosions of hydrogen, which may lead to serious accidents. In order to prevent this crisis, researchers tend to pursue new materials for fuel cladding [1,2]. The FeCrAl alloy emerged as a promising candidate for fuel cladding in nuclear power plants due to its excellent high-temperature oxidation resistance, radiation resistance, and mechanical properties [3,4,5,6,7]. People have carried out a series of studies on FeCrAl alloys. Guessev et al. [8] proposed the optimal alloy composition as Fe-(10-18)Cr-(2-6)Al-2Mo-1Nb-0.2Si-0.05Y by combining the design space of FeCrAl alloys and thermodynamic calculation. Field et al. [9,10] found that the displacement of neutrons irradiated per atom (dpa) was as high as 13.8 at a radiation temperature of 320 to 382 °C when the FeCrAl alloy was in an irradiated environment for a long time. Yamamoto et al. [11] found that with the addition of Mo and Nb, fine Fe_2_(Mo, Nb) Laves phases can be precipitated after hot rolling and annealing at 800 °C, thus improving tensile properties and thermal stability. Sun et al. [12] found that the microstructure stability of the FeCrAl alloy was closely related to the number, size, shape, and distribution of the Laves phases. Liu et al. [13] reported that Fe_2_Zr particles were formed after the Zr element was adopted, which improved the tensile strength of the alloy. However, when the trace elements are introduced in the FeCrAl alloy, the deteriorated plasticity arising from the insoluble precipitates may lead to cracks during rolling [14,15]. Consequently, it is important to explore the hot workability of the FeCrAl alloy.

The microstructure and workability of the materials during hot rolling are mainly influenced by the strain rate, deformation temperature, and strain [16]. The complex interactions of these factors under high-temperature plastic deformation are described by the constitutive equations. As a promising candidate material for nuclear fuel cladding [17,18,19], the current investigations on the hot processing map of the FeCrAl alloy are quite limited. However, it is of great significance to construct the hot processing map for investigating the hot workability of FeCrAl alloy and facilitating the practical production through hot compression [20,21,22,23].

In this paper, the hot compression tests of the FeCrAl alloy were carried out to construct the constitutive equations deriving from the flow stress–strain curves. The microstructures of the hot compressed samples were investigated. The hot processing map was constructed based on the Prasad instability criterion.

## 2. Materials and Methods

The tested material was a commercial alloy with the chemical composition of Fe-12.5Cr-4Al-0.01-Nb (wt.%).The ingot was forged after casting, and the cylindrical compression samples with a size of Φ8 × 12 mm were cut along the forging direction. The samples were compressed at 750–1200 °C with strain rates of 0.01, 0.1, 1, and 10 s^−1^ on the Gleeble-3800 thermal simulation test machine, and the deformation amount was 50%. During the compression test, the samples were heated to 1200 °C at a rate of 10 °C/s and kept for 5 min. After cooling to the required temperature of 10 °C/s and soaking for 30 s, the samples were compressed. At the same time, the data of true stress–strain curves were recorded by a computer. Then, these samples were quenched in water to retain the deformation structures. It should be noted that two 0.1 mm thick Tantalum sheets were inserted between the compression hammer and samples to reduce contact friction during the compression test. The samples were heated and compressed after vacuuming.

The microstructures at 1/4 position along the axial direction of the compressed samples were observed by an Olympus GX71 optical microscope (OM) after etching with a solution composed of FeCl_3_, HCl, and H_2_O (FeCl_3_:HCl:H_2_O = 1:10:20). The samples were subjected to electrolytic polishing with a solution composed of perchloric acid and alcohol (perchloric acid/alcohol = 1:7).Then, the electron back scatter diffraction (EBSD) equipped within a Zeiss Ultra 55 scanning electron microscope (SEM) was used to investigate the microstructures of the compressed samples.

## 3. Results and Discussion

### 3.1. Stress–Strain Curves of the FeCrAl Alloy during Compression Test

**Figure 1:**
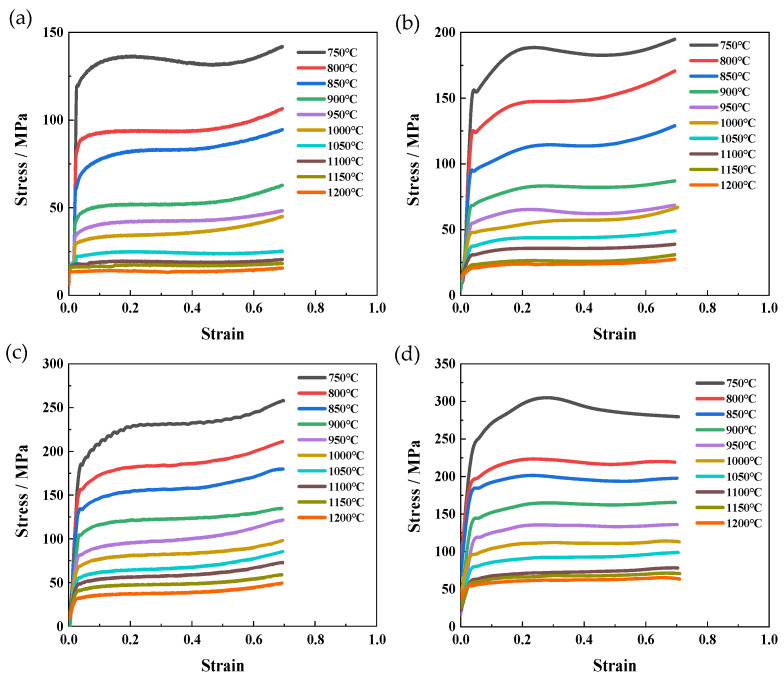
True stress–true strain curves at different deformation temperatures and the same strain rate: (**a**) 0.01 s^−1^; (**b**) 0.1 s^−1^; (**c**) 1 s^−1^; (**d**) 10 s^−1^.

The stress–strain curves under various deformation conditions (temperature and strain rate) are shown in Figure 1. These curves can be divided into three stages. Firstly, the curve increased almost linearly and reached its peak stress due to the rapidly increasing dislocation density [24]. Subsequently, the flow stress gradually decreased because of the trigger of dynamic softening. After the equilibrium was established between the work hardening and dynamic softening, the curve nearly remained constant with an increasing strain [21,25,26]. Figure 1 shows that the flow stress of the FeCrAl alloy decreased with increasing temperature or decreasing strain rate. In the case of a constant strain rate, the elevated deformation temperature enhances the thermal vibration of atoms, facilitating the dynamic recovery or recrystallization during hot compression [27]. Conversely, the increasing temperature weakens the binding force between the atoms due to expanded atom spacing. When the deformation temperature is fixed, the increased strain rate gives rise to a reduced duration of dynamic recovery or recrystallization. The work hardening may also become predominant and result in improving flow stress.

### 3.2. Regression of Constitutive Equation Parameters

The hot deformation behavior of metals can be regarded as a thermal activation process. The relationship between flow stress (*σ*), strain rate $\left(\dot{\varepsilon }\right)$, and deformation temperature (*T*) can be described by the hyperbolic sinusoidal Arrhenius relationship. including deformation activation energy (*Q*) and temperature (*T*) [28]:

(1) $$\dot{\varepsilon }=AF\left(\sigma \right)\exp[-Q/(RT)]$$

The stress function F(*σ*) in the formula has the following three different expressions:

(2) $$F\left(\sigma \right)=\sigma^{n}(\alpha \sigma <0.8)$$

(3) $$F\left(\sigma \right)=\exp\left(\beta \sigma \right)(\alpha \sigma >1.2)$$

(4) $$F\left(\sigma \right)={\left[\sinh\;(\alpha \sigma )\right]}^{n}(For\;all\;\sigma )$$

*A*, *n*, *β*, and *α* are all material constants that are independent of temperature, where *A* is the structure factor (s^−1^), *n* is the stress exponent, *β* = *nα*, and *α* is the stress factor (MPa^−1^); *Q* is thermal deformation activation energy (kJ·mol^−1^), also known as dynamic softening activation energy, which reflects the equilibrium relationship between work hardening and dynamic softening during thermal deformation; *R* is the gas constant (8.3145 J·mol^−1^·K^−1^); $\dot{\varepsilon }$ is the strain rate (s^−1^); *σ* is flow stress; and *T* is the thermodynamic temperature (K).

Substitute Equations (2) and (3) into Equation (1), respectively, and logarithm both sides of the equation to obtain the following formula:

(5) $$ln\dot{\varepsilon }=lnA_{1}+n_{1}ln\sigma -Q/(RT)$$

(6) $$ln\dot{\varepsilon }=lnA_{2}+\beta \sigma -Q/(RT)$$

*A*_1_ and *n*_1_ are the structural factor (s^−1^) and stress exponent at low stress levels, respectively; *A*_2_ is the structural factor (s^−1^) at high stress levels.

**Figure 2:**
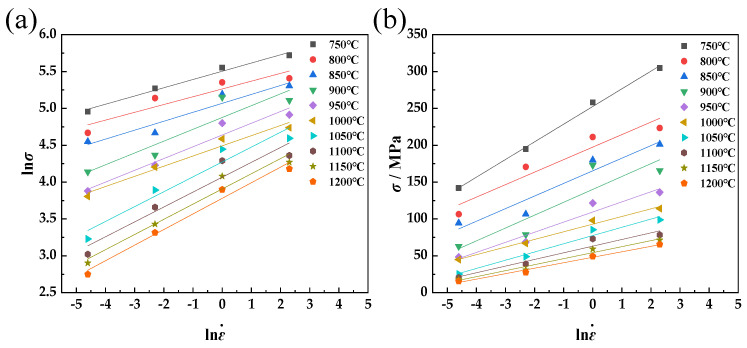
Relationships between ln*σ* and ln$\dot{\varepsilon }$ (**a**) and *σ* and ln$\dot{\varepsilon }$ (**b**).

By substituting the true stress–true strain data into the above formula, the fitting curves of ln*σ*-ln$\dot{\varepsilon }$ and σ-ln$\dot{\varepsilon }$ can be obtained, as shown in Figure 2. Linear regression is carried out with the least square method, and the constants at different temperatures are obtained: *n*, *β*, and *α*. The reciprocal of the average slope of the straight line obtained by fitting ln*σ* with ln$\dot{\varepsilon }$ is the parameter *n* = 6.1772, and the reciprocal of the average slope of the straight line obtained by fitting σ with ln$\dot{\varepsilon }$ is the parameter *β* = 0.0738, *α* = *β*/*n*, so *α* = 0.0119.

**Figure 3:**
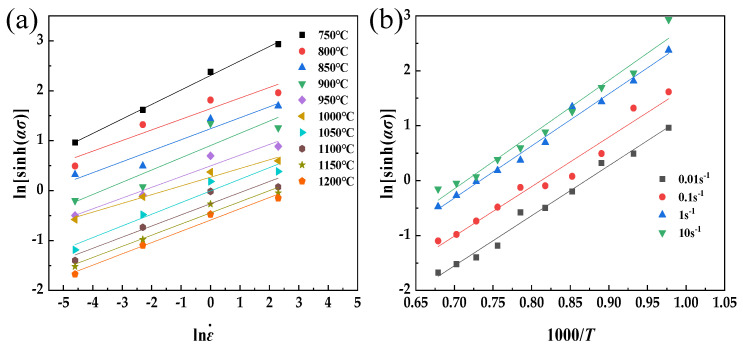
Relationships between ln[sinh(ασ)] and ln$\dot{\varepsilon }$ (**a**) and ln[sinh(*ασ*)] and 1000/*T* (**b**).

After determining the value of *α*, *n* undergoes further modification by substituting Equation (4) into Equation (1) and subsequently taking the natural logarithm on both sides. The modified *n* is found to be 4.2293, and the derivation of both sides of the equation results in a functional relationship of ln[sinh(*ασ*)] − 1000/*T*. This relationship yields a linear function, as shown in Figure 3 below. When the strain rate is constant, the thermal deformation activation energy *Q* of FeCrAl alloys can be determined using the modified *n*. A linear fit to a straight line is obtained by fitting ln[sinh(*ασ*)] to 1000/*T* (where *T* is the Kelvin temperature), with an average slope value of 9.37. Therefore, the thermal deformation activation energy of the material can be calculated at 329.49 kJ/mol by Equation (7).

(7) $$Q=R\frac{\partial ln\dot{\varepsilon }}{\partial ln\left[sinh\left(\alpha \sigma \right)\right]}\frac{\partial ln\left[sinh\left(\alpha \sigma \right)\right]}{\partial T^{-1}}$$

For all stress levels, substituting Equation (4) into Equation (1) can be expressed as follows:

(8) $$\dot{\varepsilon }={\left[Asinh\left(\alpha \sigma \right)\right]}^{n}\exp[-Q/RT]$$

Simultaneously, the strain rate of metals during thermoplastic deformation is governed by a thermal activation process. The relationship between the strain rate ($\dot{\varepsilon }$) and the deformation temperature (*T*) is aptly expressed through the Zener–Hollomon parameter (*Z* parameter), as elucidated by Zener and Hollomon in their theoretical framework [29,30]. The Zener–Hollomon parameter is defined as follows:

(9) $$Z=\dot{\varepsilon }\exp\left[Q/(RT\right)]=A{\left[\sinh(\alpha \sigma )\right]}^{n}$$

**Figure 4:**
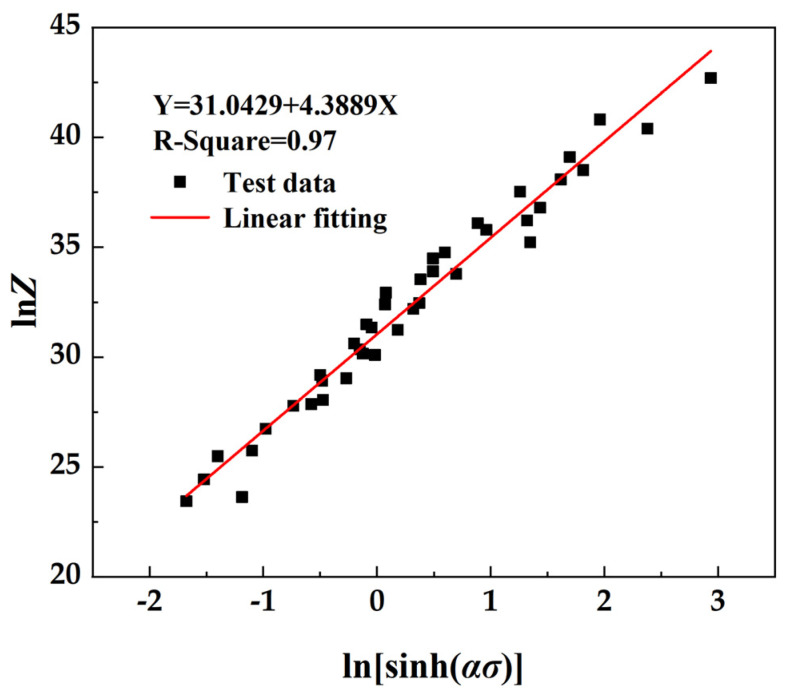
Relationship between ln*Z* and ln[sinh(*ασ*)].

The relationship between ln*Z* and ln[sinh(*ασ*)] is derived by taking the logarithm of both sides of the equation and substituting the correlation constant along with the true stress–true strain data obtained from hot compression experiments. This relationship is depicted in Figure 4 below, where *σ* represents the peak stress corresponding to various process parameters during hot deformation.

The ln*Z*-ln[sinh(*ασ*)] function relationship obtained is linear. By substituting *Q*, *n*, and *α* into Equation (8), the constitutive equation of flow stress during hot compression deformation can be obtained, and the expression of the *Z* parameter can be obtained. From the fitting graph of ln*Z* and ln[sinh(*ασ*)], the intercept is ln*A*, so *A* = 3.032 × 10^13^ s^−1^. By substituting Equation (8), the constitutive equation of the material is $\dot{\varepsilon }$ = 3.032 × 10^13^[sinh(0.0119 *σ*)]^4.2293^exp[−329.49/*RT*].

**Figure 5:**
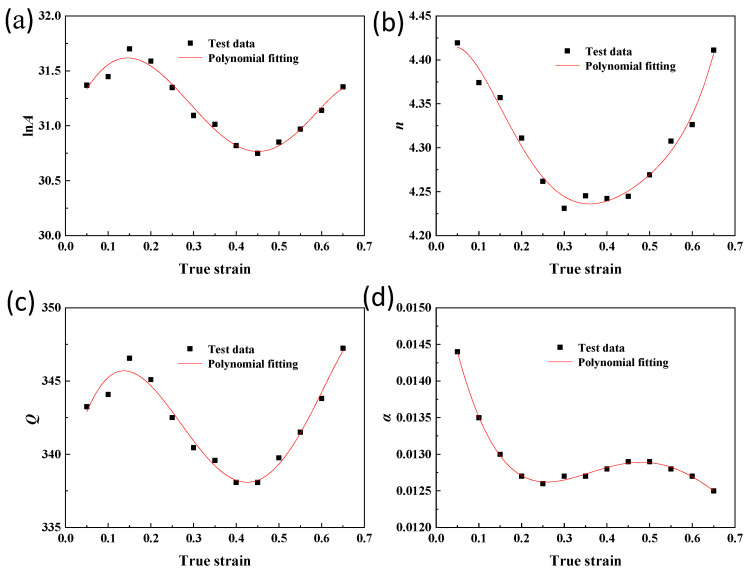
Variation of material constants with true strain: (**a**) ln*A*; (**b**) *n*; (**c**) *Q*; (**d**) *α*.

**Figure 6:**
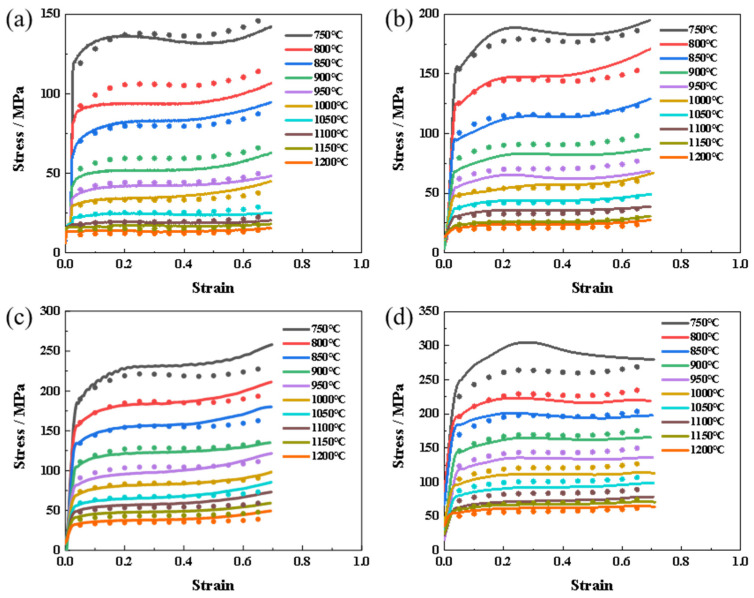
Comparison of experimental and predicted stress–strain values for FeCrAl alloys at different temperatures and strain rates: (**a**) 0.01 s^−1^; (**b**) 0.1 s^−1^; (**c**) 1 s^−1^; (**d**) 10 s^−1^. (Dots represent the test data, while lines display the polynomial fitting).

In the actual plastic deformation process, the flow behavior of metallic materials at high temperatures is influenced by the strain. The constitutive equations presented earlier are established based on peak stress (*σ*) for various process parameters in the hot deformation process without considering the effect of strain. The values of material constants stabilize gradually with an increase in true strain. Consequently, it becomes necessary to develop strain-compensated equations for FeCrAl alloys. This study calculates the stresses corresponding to the strains ranging from 0 to 0.65 in sequential intervals of 0.05. The material constants ln*A*, *n*, *Q*, and *α* are solved based on these equations, and a fifth-order polynomial is fitted to each parameter. Consequently, a fifth-order polynomial of material constants with respect to the strain compensation constitutive equation is established, as shown in Equation (10), with the corresponding coefficients listed in Table 1. The fitted variations of different material constants with strain are illustrated in Figure 5. By combining this constitutive equation with Equation (10), stress values can be calculated for different deformation conditions. To validate the accuracy of the strain-compensated constitutive equation, experimental values of peak stresses under various thermal deformation conditions are compared with calculated values obtained from the constitutive equation. The results in Figure 6 demonstrate that the calculated stress values aligned well with the experimental values. Thus, the developed equation accurately predicts the flow stresses of FeCrAl alloys across a range of deformation conditions.

$$lnA=a_{0}+a_{1}\varepsilon +a_{2}\varepsilon^{2}+a_{3}\varepsilon^{3}+a_{4}\varepsilon^{4}+a_{5}\varepsilon^{5}$$

$$n=b_{0}+b_{1}\varepsilon +b_{2}\varepsilon^{2}+b_{3}\varepsilon^{3}+b_{4}\varepsilon^{4}+b_{5}\varepsilon^{5}$$

$$Q=c_{0}+c_{1}\varepsilon +c_{2}\varepsilon^{2}+c_{3}\varepsilon^{3}+c_{4}\varepsilon^{4}+c_{5}\varepsilon^{5}$$

(10) $$\alpha =d_{0}+d_{1}\varepsilon +d_{2}\varepsilon^{2}+d_{3}\varepsilon^{3}+d_{4}\varepsilon^{4}+d_{5}\varepsilon^{5}$$

**Figure 7:**
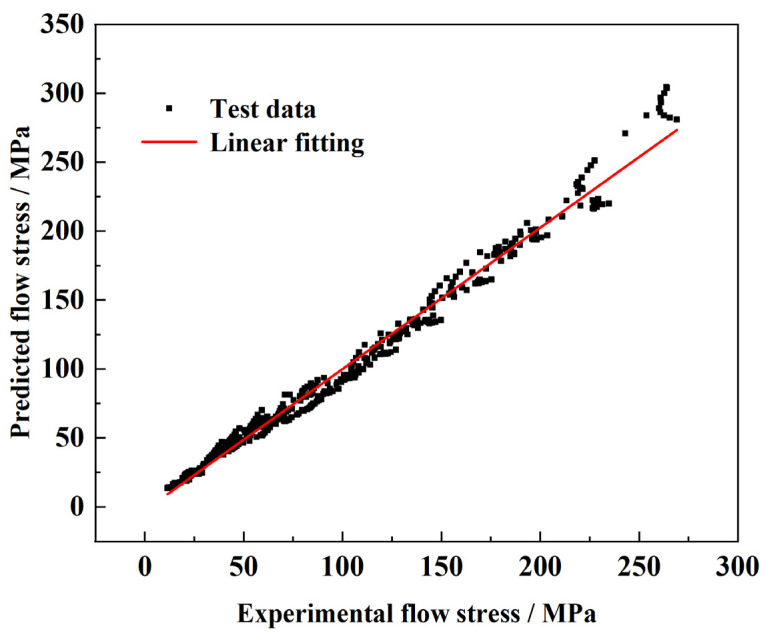
Correlation between experimental and predicted flow stresses at chosen deformation conditions.

To validate the accuracy of the strain-compensated constitutive equation, the experimental values of peak stresses under various deformation conditions were compared with the calculated values. Figure 7 shows the correlation between the predicted stress and the test stress after linear fitting. The square correlation coefficient R^2^ of the test value and the predicted value was up to 0.987, indicating that the predicted data have a good correlation with the test data.

In order to further evaluate the accuracy of the established constitutive equation, the average relative error (Δ, %) between the prediction and the test stress was calculated. The calculation parameter expression is shown in Equation (11). Where E_i_ is the test value, MPa; P_i_ is the predicted value, MPa; and N is the number of total data points. The calculation results showed that the prediction error of the constitutive equation is 7.23% in the whole range of deformation parameters. Thus, the developed equation accurately predicted the flow stresses of FeCrAl alloys across a range of deformation conditions.

(11) $$∆=(1/N)\sum_{i=1}^{N}\left|(E_{i}-P_{i})/P_{i}\right|\times 100\%$$

### 3.3. Construction of Hot Processing Maps

The hot processing map of three typical strains (reduction 20%, 30%, and 50%, corresponding true strains *ε* = 0.22, 0.36, and 0.69, respectively) was constructed for an FeCrAl alloy. The subsequent production, development, and utilization of FeCrAl alloys are linked to early hot working technology. One of the best methods to investigate this technology is hot processing map [31,32,33,34]. These maps offer an opportunity to control microstructure evolution during hot deformation and to analyze the deformation mechanism. In addition, the easily forming intervals and unstable forming intervals are illustrated, aiding in avoiding low-efficiency zones and reducing defects. Consequently, the hot processing maps serve as important theoretical foundations for industrial production. The hot processing maps were conducted using the experimental data, the dynamic material model theory, and Prasad stability criterion [35].

The hot processing map consists of two components: the power dissipation map and the instability map. According to the dynamic material model, the absorbed energy is primarily allocated to plastic deformation and microstructure evolution. The former is denoted as power dissipation (*G* component), while the latter is power dissipation coquantity (*J* component). The ratio of these components can be expressed through the strain rate sensitivity index [35],

(12) $$m=\frac{\partial J}{\partial G}=\frac{\int_{0}^{\sigma }\dot{\varepsilon }d\sigma }{\int_{0}^{\dot{\varepsilon }}\sigma d\dot{\varepsilon }}$$

When the material is in the most ideal linear dissipation state under thermal deformation, that is, *m* = 1, the dissipation coquantity J will reach the maximum value of *J*_max_ =$\frac{\sigma \cdot \dot{\varepsilon }}{2}$. For the nonlinear dissipation state, the energy consumption efficiency can be expressed by introducing the efficiency of power dissipation (*η*):

(13) $$\eta =\frac{J}{J_{max}}=\frac{2m}{m+1}$$

By taking the logarithm of flow stress *σ* and strain rate $\dot{\varepsilon }$ under specific strain *ε* and temperature *T* measured by hot compression experiments and fitting with a cubic polynomial, we can achieve the following:

(14) $$lg\sigma =a+blg\dot{\varepsilon }+c{\left(lg\dot{\varepsilon }\right)}^{2}+d{\left(lg\dot{\varepsilon }\right)}^{3}$$

where a, b, and c are constants and a ≠ 0, the strain rate sensitivity index *m* can be obtained by the following formula:

(15) $$m=\frac{\partial (lg\sigma )}{\partial (lg\dot{\varepsilon })}=b+2clg\dot{\varepsilon }+3d{\left(lg\dot{\varepsilon }\right)}^{2}$$

The power dissipation factor (*η*) can be determined by substituting the calculated value of *m* into the aforementioned formula. The power dissipation map illustrates the correlation between *η*, deformation temperature, and deformation rate. Typically, a higher-power dissipation factor indicates greater energy utilized for microstructure evolution while concurrently dissipating more energy in the form of heat during plastic deformation. In the power dissipation map, a larger value is considered optimal for the processing zone.

However, it is crucial to note that potential issues such as cracking and void formation during hot working can significantly impact the power dissipation factor. Therefore, judging the optimal processing interval solely based on *η* values may be insufficient. Prasad et al. established criteria for determining the plastic instability zone, considering the critical condition of rheological instability and adhering to the principles of irreversible thermodynamic extremum [35].

(16) $$\xi \left(\dot{\varepsilon }\right)=\frac{\partial \mathrm{lg}(\frac{m}{m+1})}{\partial lg\dot{\varepsilon }}+m=\frac{1}{2.3m(m+1)}\cdot \frac{\partial m}{\partial lg\dot{\varepsilon }}+1$$

By differentiating both sides of the aforementioned formula, (∂*m*/$\partial lg\dot{\varepsilon }$) can be obtained. A Prasad instability coefficient less than 0 indicates a microstructure undergoing an instability transition during hot working. The shadowed area in the map represents values less than 0, constituting the instability map.

**Figure 8:**
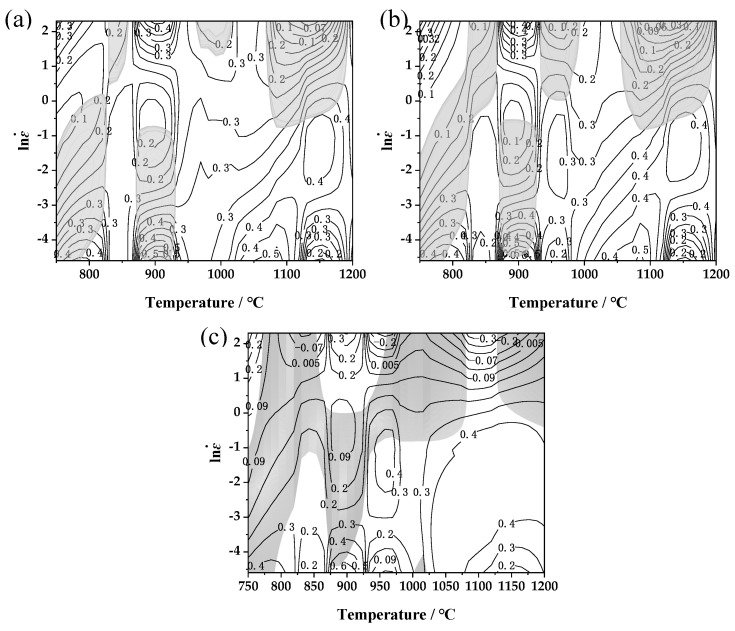
Hot processing map under different true strains: (**a**) 0.22; (**b**) 0.36; (**c**) 0.69. (Grey shadow represents the instability region).

In conclusion, the power dissipation map and the instability map are overlaid to create the hot working map, depicted in Figure 8. The contour values in the map signify the power dissipation factor (*η*), with the shaded portion indicating the region where the instability coefficient $\xi (\dot{\varepsilon })$ is less than 0, signifying the instability $\xi (\dot{\varepsilon })$.

As can be seen from Figure 8, as the true strain increases from 0.22 to 0.69, the instability region of the material is merged from five small regions into two large regions, the overall shape does not change much, and the maximum power dissipation increases from 0.5 to 0.6. Analyzing the hot processing map for the material with a true strain of 0.69 revealed the distinct instability zones across low-, medium-, and high-temperature ranges. Instability zone I: temperature range of 750–870 °C; strain rate of 0.01–10 s^−1^. A small stability zone exists within this temperature range. The dissipation value was low; the microstructure was mainly characterized by dynamic recovery; and the narrow temperature range was not conducive to efficient production. Instability zone II: temperature range of 870–930 °C; strain rate of 0.01–1 s^−1^. Instability zone III: temperature range of 950–1080 °C; strain rate of 0.4–10 s^−1^. Instability zone VI: temperature range of 1130–1200 °C; strain rate of 0.7–10 s^−1^. Stable regions are primarily concentrated in the medium-high, moderate, and low strain ranges. Stable zone I: temperature range of 930–1000 °C; strain rate of 0.05–0.4 s^−1^. Stable zone II: temperature range of 1050–1200 °C; strain rate of 0.01–0.4 s^−1^. This region exhibited a high power dissipation value, conducive to the dynamic recrystallization of the microstructure. The processing temperature window is larger, making it the optimal processing region for this material.

### 3.4. Microstructures of the Hot Compressed Samples

**Figure 9:**
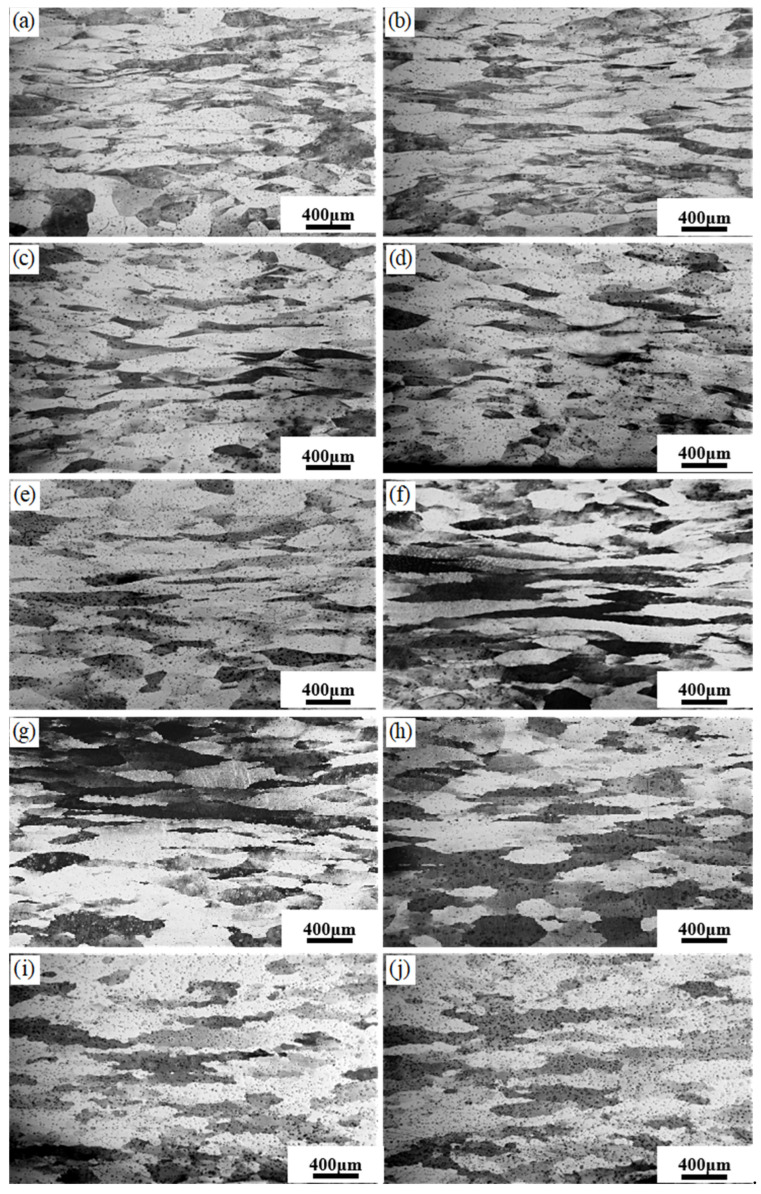
Metallographic structure at different temperatures with deformation rate of 0.1 s^−1^: (**a**) 750 °C; (**b**) 800 °C; (**c**) 850 °C; (**d**) 900 °C; (**e**) 950 °C; (**f**) 1000 °C; (**g**) 1050 °C; (**h**) 1100 °C; (**i**) 1150 °C; (**j**) 1200 °C.

**Figure 10:**
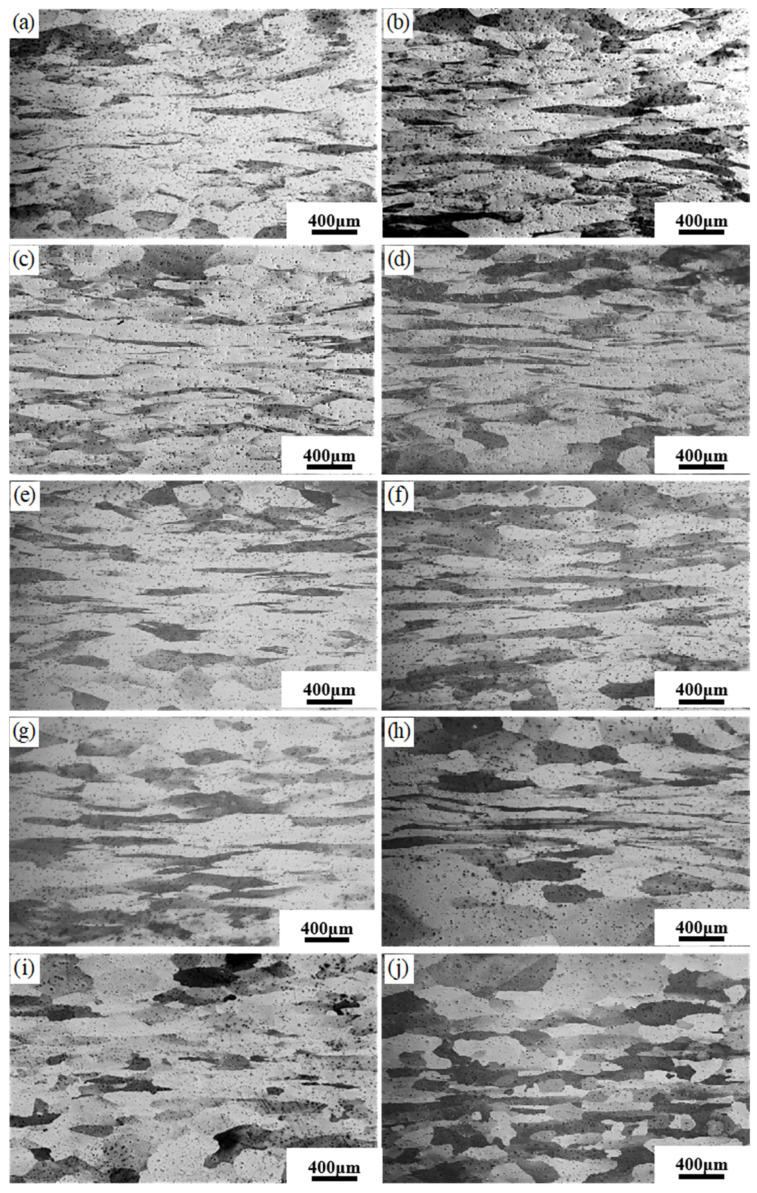
Metallographic structure at different temperatures with deformation rate of 1 s^−1^: (**a**) 750 °C; (**b**) 800 °C; (**c**) 850 °C; (**d**) 900 °C; (**e**) 950 °C; (**f**) 1000 °C; (**g**) 1050 °C; (**h**) 1100 °C; (**i**) 1150 °C; (**j**) 1200 °C.

Figure 9 shows the microstructure of the hot compressed sample at a strain rate of 0.1 s^−1^. Most grains in the 750 °C and 800 °C samples were elongated along the direction of material flow due to the deformation. With the temperature rising to 1000 °C, the bended grain boundaries (grain boundary arching phenomenon) indicated the occurrence of discontinuous dynamic recrystallization. In cases of deformation at 1150 °C and 1200 °C, a large number of fine grains emerged due to dynamic recrystallization. Figure 10 showed the microstructure of the sample deformed at 1 s^−1^. Because of the increased strain rate, the duration of dynamic recrystallization has decreased and may be insufficient for dynamic recrystallization. As a result, the grain boundary arched at 1200 °C-0.1 s^−1^, while the equiaxed grains formed at 1200 °C-1 s^−1^.

**Figure 11:**
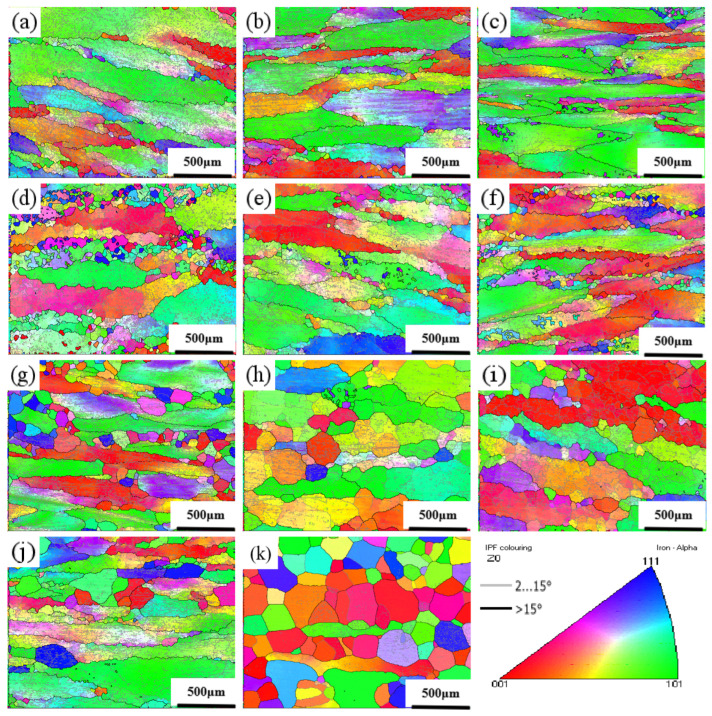
Crystal orientation diagram at different temperatures and different deformation rates: (**a**) 900 °C-0.01 s^−1^; (**b**) 950 °C-0.1 s^−1^; (**c**) 950 °C-1 s^−1^; (**d**) 1000 °C-0.01 s^−1^; (**e**) 1050 °C-0.1 s^−1^; (**f**) 1050 °C-1 s^−1^; (**g**) 1050 °C-10 s^−1^; (**h**); 1150 °C-0.01 s^−1^ (**i**) 1150 °C-0.1 s^−1^; (**j**) 1150 °C-1 s^−1^; (**k**) 1150 °C-10 s^−1^.

Figure 11 shows the orientation image maps of the samples. The samples exhibited the typical dynamic recovery microstructures with dense low-angle (≤15°) grain boundaries when the deformation temperature was no more than 950 °C, even at a low strain rate of 0.01 s^−1^. When the temperature was improved to 1000 °C, many small grains emerged at a strain rate of 0.01 s^−1^ because of the dynamic recrystallization. When the temperature increased to 1050 °C and the strain rate was above 0.01 s^−1^, the recrystallization was restricted. When the temperature rose to 1150 °C, the dynamic recrystallization occurred at all four strain rates (0.01 s^−1^, 0.1 s^−1^, 1 s^−1^, and 10 s^−1^). When the strain rate was improved to 10 s^−1^, grain growth was inhibited due to insufficient time for dynamic recrystallization. Thus, all the new recrystallization grains were small and equiaxed.

As shown in Figure 11b, the sample exhibited a typical dynamic recovery microstructure mainly composed of elongated grains, and the power dissipation value was 30–40%. These characteristics corresponded to the stable zone I in the hot processing maps. Similarly, a small number of dynamic recrystallized grains were distributed along the grain boundaries in Figure 11g, while the corresponding power dissipation value of this deformation condition was 7%. These corresponded to instability zone III in the hot processing maps. As shown in Figure 11i, a typical continuous recrystallization microstructure composed of a large number of serrated recrystallized grains at grain boundaries was formed, and the power dissipation value was 40%, corresponding to the optimal stability zone II in the hot processing maps.

## 4. Conclusions

In this work, the microstructure evolution and hot deformation behavior of an FeCrAl alloy were investigated in a wide temperature and strain rate range. The hot processing maps were established, and the optimal hot working ranges were given. Based on this study, the following conclusions can be drawn:

(1) The flow stress of FeCrAl alloys decreased with increasing deformation temperatures and increased with an improving strain rate. The thermal deformation activation energy was determined to be 329.49 kJ/mol. The resulting constitutive equation, considering strain compensation, is expressed as $\dot{\varepsilon }\;$= 3.032 × 10^13^[sinh(0.0119σ)]^4.2293^ exp [−329.49/RT].
(2) The hot processing map revealed four small instability zones: Instability zone I: temperature of 750–870 °C; strain rate of 0.01–10 s^−1^. Instability zone II: temperature of 870–930 °C; strain rate of 0.01–1 s^−1^. Instability zone III: temperature of 950–1080 °C; strain rate of 0.4–10 s^−1^. Instability zone VI: temperature of 1130–1200 °C; strain rate of 0.7–10 s^−1^. The optimum processing range is 1050–1200 °C with a strain rate of 0.01–0.4 s^−1^.
(3) At lower temperatures (750 °C and 800 °C) and strain rates of 0.1 s^−1^ and 1 s^−1^, the microstructures remained in a deformation state. As the temperature increased to 1000 °C, the bended grain boundaries emerged due to dynamic recrystallization. With further increasing temperatures, lots of recrystallized grains were formed. The microstructure characteristics and related power dissipation values under various conditions corresponded well with the stability and instability zones in the hot processing map.

## Figures and Tables

**Table 1:** Coefficient values of fifth-order polynomials in Equation (9).

ln*A*	*n*	*Q*	*α*
a_0_ = 30.95279	b_0_ = 4.39193	c_0_ = 338.10486	d_0_ = 0.01573
a_1_ = 9.17155	b_1_ = 1.15911	c_1_ = 123.31095	d_1_ = −0.03196
a_2_ = −27.80001	b_2_ = −17.00512	c_2_ = −574.27089	d_2_ = 0.11212
a_3_ = −57.92418	b_3_ = 60.54788	c_3_ = 470.3829	d_3_ = −0.15243
a_4_ = 244.31678	b_4_ = −88.55523	c_4_ = 898.15577	d_4_ = 0.06349
a_5_ = −185.57526	b_5_ = 48.49286	c_5_ = −1017.49623	d_5_ = 0.00603

## Data Availability

The data presented in this study are available on request from the corresponding author and the first author (due to privacy).
